# Isthmic spondylolisthesis combined with schwannoma occurring at the same vertebral level: a case report and literature review

**DOI:** 10.3389/fsurg.2025.1457408

**Published:** 2025-02-20

**Authors:** Renrui Niu, Jianhui Zhao, Chaoyuan Li, Fengshuo Guo, Yuchi Duan, Wenqi Luo, Rui Gu

**Affiliations:** Department of Spine Surgery, China-Japan Union Hospital of Jilin University, Changchun, China

**Keywords:** isthmic spondylolisthesis, responsible lesion, schwannoma, radiculopathy, treatment

## Abstract

**Background:**

The occurrence of schwannomas at the level of isthmic spondylolisthesis has not yet been reported. Preoperative identification of the responsible lesion and a rational surgical plan are essential for successful surgery.

**Case presentation:**

We report the case of a 56-year-old woman who presented with a six-year history of low back pain and a three-year history of radiating pain in the left lower extremity. Physical examination revealed signs of left L5 root compression. Computed tomography revealed left L5–S1 intervertebral foramen stenosis with an isthmic fracture. Unexpectedly, magnetic resonance imaging (MRI) showed an abnormal 12 mm*11 mm*21 mm intradural mass with inhomogeneous contrast enhancement at the level of the spondylolisthesis. Isthmic spondylolisthesis and schwannoma were suspected. Based on the imaging and physical findings, we inferred that the lower-extremity pain was primarily caused by lumbar spondylolisthesis. Under general anesthesia, the patient underwent posterior lumbar interbody fusion of L5–S1 and intradural total tumorectomy. Histopathological examination of the surgical specimen revealed a schwannoma. The patient's symptoms resolved postoperatively, and intervertebral fusion was satisfactory at the 12-month follow-up.

**Conclusion:**

This case demonstrates the difficulty of determining the responsible lesion, highlighting the importance of meticulous clinical and imaging examinations. Determining the responsible lesion is crucial for diagnosis and treatment.

## 1 Introduction

Preoperative identification of the responsible lesion is crucial for effective surgical intervention (1). Schwannoma is a common benign tumor of the nervous system, with pain being the most common initial symptom. The male-to-female ratio of patients with schwannoma is 72.3% vs. 27.7% and it frequently occurs at T12–L5 (2). Isthmic spondylolisthesis can be defined as the anterior translation of a vertebral body relative to the one subjacent to it and secondary to an abnormality of the pars interarticularis (3). According to Sakai's (4) research, the prevalence of spondylolysis in men is markedly higher than that in women in the Japanese general population. Spondylolysis often leads to low back pain and radicular symptoms in the lower extremities. Single schwannomas or lumbar spondylolistheses are easily identified and treated by surgeons (3). In Sattam's (5) report of 634 patients with low back pain, 2.7% had significant pathologies other than disc disease; the incidence of schwannoma in patients with isthmic spondylolisthesis is relatively low. Identifying the responsible lesion and managing it during surgery poses significant challenges.

Here, we present a case of spondylolisthesis and schwannoma at the same vertebral level with radiating pain in the left lower extremity. Surgical treatment after determining the responsible lesion by imaging and physical examination yielded good results. We also provide a comprehensive literature review to emphasize that identifying the responsible lesion is essential for a successful operation.

## 2 Case presentation

**Figure 1:**
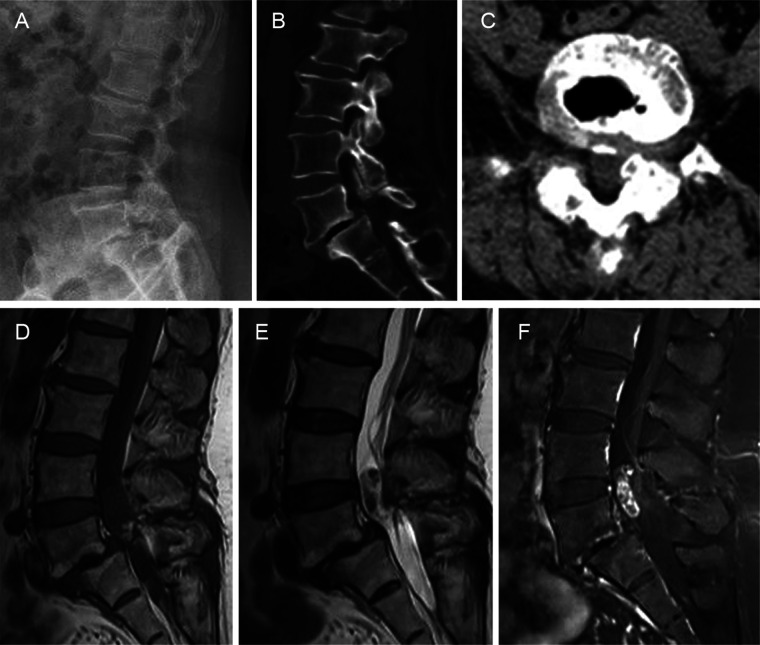
Preoperative imaging. **(A)** Radiographs of the lumbar spine showing grade II L4–5 spondylolisthesis. **(B,C)** Computed tomography images showing left L5–S1 intervertebral foramen stenosis and the isthmic fracture. **(D,E)** Magnetic resonance image shows equal or low signals on the T1-weighted imaging sequence and high signals on the T2-weighted imaging sequence. **(F)** Enhanced scan showing inhomogeneous enhancement.

**Figure 2:**
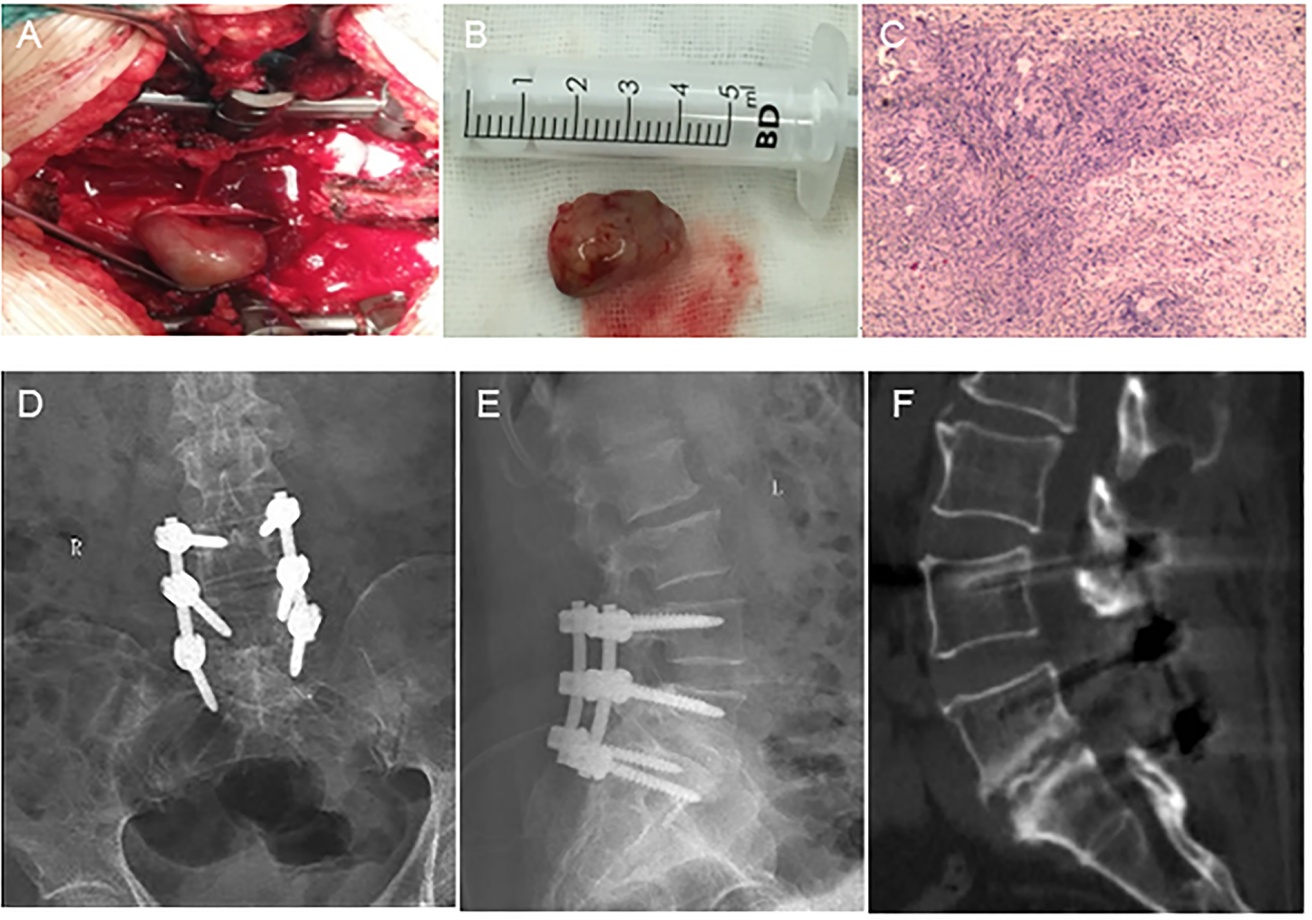
Intraoperative, pathological, and postoperative images. **(A)** The tumor is located in the intradural region. **(B)** The tumor has a solid and oval complete capsule. **(C)** Low-magnification (×40) hematoxylin-and-eosin-stained section showing typical Antoni A hypercellular areas composed of spindle cells arranged in fascicular and sheet-like patterns with nuclear palisading (circles). **(D,E)** Interbody vertebrae completely fused. **(F)** L5–S1 intervertebral fusion is satisfactory.

A 56-year-old woman presented to our department with a six-year history of low back pain and a three-year history of radiating pain in the left lower extremity. The low back pain began six years ago with no apparent cause and gradually worsened over three years, accompanied by radiating pain and numbness in the left lower extremity. The patient had taken medication and undergone conservative treatments such as massage and physiotherapy, but her symptoms had not improved. The patient had no history of hypertension, diabetes, heart disease, tumors, or infectious diseases. There was a palpable “step” at L4–5 that was accompanied by tenderness at L4–5 and L5–S1. The pain worsened upon hyperextension of the left lower limb and radiated from the posterolateral thigh to the lateral gastrocnemius muscle. Hypesthesia was observed over the lateral aspect of the left gastrocnemius muscle and the dorsum of the left foot. Radiographs of the lumbar spine showed grade II spondylolisthesis at L5–S1. Computed tomography (CT) revealed left L5–S1 intervertebral foramen stenosis with an isthmic fracture. Magnetic resonance imaging (MRI) revealed equal or low signal intensity on T1-weighted images and high signal intensity on T2-weighted images. Contrast-enhanced scans revealed inhomogeneous enhancement (Figure 1). Posterior lumbar interbody fusion and intradural tumorectomy were also performed at L5–S1. The schwannoma reached up to the posterior aspect of the L4 vertebral body and down to the posterior aspect of the L5 vertebral body; we removed the majority of the L4 vertebral spinous processes and segmental lamina. In addition, this was a menopausal woman with lumbar spondylolisthesis. To prevent pedicle screw extraction during repositioning and to reduce stresses on the L5 pedicle screw, we stabilized and extended the fusion to the L4 vertebrae. During the procedure, we used neuromonitoring together with PLIF procedure for decompression of the L5 nerve root. In addition, we used head-mounted light sources and microscopes to make sure the schwannoma was removed thoroughly and to prevent nerve damage. Intraoperatively, scar tissue was confirmed in the L5 isthmus and severe adhesion to the left L5 nerve root was found. The tumor was removed by gross total resection (Figures 2A,B), further confirming schwannoma by hematoxylin and eosin staining (Figure 2C). The patient did not experience lower back pain or radiating pain to the lower extremities postoperatively. Five days after surgery, the patient could sit and walk with the assistance of a waist support. At the 12-month follow-up, the intervertebral bodies were completely fused. The patient reported significant relief of low back pain and lower limb pain, and she was able to perform normal daily activities (Figures 2D–F).

## 3 Results and discussion

This report describes an extremely rare case of isthmic spondylolisthesis combined with schwannoma occurring at the same vertebral level. The patient was successfully treated with a single-stage surgery due to definitive lesion diagnosis. This highlights the importance of accurate diagnosis for disease outcome. Table 1 summarizes cases of lumbar spondylolisthesis combined with spinal tumor or neural tumor in which surgical treatment resulted in favorable outcomes after identifying the responsible lesion (6–10). None of these cases were successfully treated without a definitive diagnosis. All of these cases emphasize the importance of defining the diagnosis of the responsible lesion, as effective treatment outcomes are closely correlated with the diagnosis.

**Table 1:** Cases of lumbar spondylolisthesis combined with a bone or nerve tumor.

Disease 1	Disease 2	Age	Sex	Author	Treatment	Outcome	Ref.
L4–5 spondylolisthesis	Dural ectasia	32	Female	A. H. Bensaid,	Not mentioned	Not mentioned	(6)
Spondylolisthesis	Inflammatory myofibroblastic tumor	56	Female	Sang Hoon Yoon	Posterior decompression followed by arachnoid web removal	Intermittent claudication gone, paresthesia on right toes remained.	(7)
L5–S1 spondylolisthesis	Neurofibromatosis	26	Female	Barry Cheaney II	Anterior lumbar interbody fusion (ALIF)	Complete resolution of preoperative symptoms	(8)
L5–S1 spondylolisthesis	Paraganglioma	69	Female	Ilya Laufer	Foraminotomies and lumboiliac fusion	Lower-extremity strength improved	(9)
Spondylolysis/spondylolisthesis	6 benign tumors, 2 primary malignant tumors, 6 secondary or systemic tumors	47	5 Male, 9 Female	Riccardo Cecchinato	En bloc resection, vertebroplasty, local corticoid injection, and intralesional excision	Not mentioned	(10)

Isthmic spondylolisthesis occurring in combination with schwannoma at the same spinal level has been rarely reported. The formation of isthmic spondylolisthesis is believed to be a multifactorial process. First, the pars interarticularis of isthmic spondylolisthesis is subject to high stress due to the lumbosacral junction. In addition, the pars in the lower lumbar spine is relatively thin. A weak pars combined with increased forces concentrated on the pars during lumbar extension or rotation can lead to stress fractures (11, 12). Schwannoma, also known as neurilemmoma, is a benign tumor that originates from Schwann cells, which are derived from the neural crest (13). Therefore, it is a coincidence in this patient that isthmic spondylolisthesis combined with schwannoma at the same vertebral level. Isthmic spondylolisthesis often requires fusion without decompression or with indirect decompression (14). For patients with schwannoma, laminectomy + microscopic excision + pedicle screw fixation has the highest cure rate (15). After a review of the literature, we found that Wada (16) reported an 89-year-old patient with an intradural extramedullary tumor and cervical spondylosis. Kaplan (17) reported a patient with neurilemmoma of the cauda equina and spondylolisthesis. Neither of the two cases reported the treatment and diagnosis of different diseases occurring at the same spinal level. Naturally, the diseases responsible for the patient's symptoms are associated with similar characteristics, making it challenging to identify the culprit lesion. The patient had hypoesthesia over the lateral aspect of the left calf and foot dorsum, and the extensor hallucis longus muscle strength was Class IV. These manifestations were consistent with those of L5–S1 intervertebral foramen stenosis. We believe that isthmic spondylolisthesis (degree II) caused these symptoms. Therefore, we performed lumbar spondylolisthesis surgery with incision and internal fixation, along with nerve sheath tumor removal. Lower back pain and radiating pain in the lower extremities disappeared immediately after surgery, and the 12-month follow-up showed effective treatment outcomes. There are still some limitations in this report: (1) One case alone cannot provide sufficient empirical guidance on the diagnosis and treatment of such diseases; and (2) There was insufficient follow-up time to provide long-term prognostic patient outcomes.

In the present case, we identified the lesion responsible for isthmic spondylolisthesis and achieved gross total resection with posterior decompression and fusion in a single-stage operation for the following reasons. First, due to the large size of the tumor, simple decompression and fusion may have caused neurological deficits (18, 19). Second, isolated tumor resection could have caused local scar formation (20), making decompression and fusion more difficult. Third, several studies have shown that gross total resection is associated with a low risk of recurrence (21–23). Therefore, we preferred to perform these two procedures concomitantly.

## 4 Conclusions

Our experience with this case shows that determining the responsible lesion is essential; therefore, meticulous attention should be paid to clinical and imaging examinations. The identification of the responsible lesion is a prerequisite for surgical efficacy. This case serves to remind spine surgeons that signs and imaging are important for accurate diagnosis, which is a prerequisite for a good result.

## Data Availability

The raw data supporting the conclusions of this article will be made available by the authors, without undue reservation.
